# G protein-coupled receptor kinase 2 promotes cardiac hypertrophy

**DOI:** 10.1371/journal.pone.0182110

**Published:** 2017-07-31

**Authors:** Philipp Schlegel, Julia Reinkober, Eric Meinhardt, Henrike Tscheschner, Erhe Gao, Sarah M. Schumacher, Ancai Yuan, Johannes Backs, Patrick Most, Thomas Wieland, Walter J. Koch, Hugo A. Katus, Philip W. Raake

**Affiliations:** 1 Department of Internal Medicine III, Cardiology, University Hospital Heidelberg, University of Heidelberg, Heidelberg, Germany; 2 DZHK (German Centre for Cardiovascular Research), Partner Site Heidelberg/Mannheim, Germany; 3 Center for Translational Medicine, Department of Pharmacology, Temple University School of Medicine, Philadelphia, Pennsylvania, United States of America; 4 Department of Molecular Cardiology and Epigenetics, University of Heidelberg, Heidelberg, Germany; 5 Institute of Experimental and Clinical Pharmacology and Toxicology, University of Heidelberg, Medical Faculty Mannheim, Mannheim, Germany; Cleveland Clinic, UNITED STATES

## Abstract

The increase in protein activity and upregulation of G-protein coupled receptor kinase 2 (GRK2) is a hallmark of cardiac stress and heart failure. Inhibition of GRK2 improved cardiac function and survival and diminished cardiac remodeling in various animal heart failure models. The aim of the present study was to investigate the effects of GRK2 on cardiac hypertrophy and dissect potential molecular mechanisms. In mice we observed increased GRK2 mRNA and protein levels following transverse aortic constriction (TAC). Conditional GRK2 knockout mice showed attenuated hypertrophic response with preserved ventricular geometry 6 weeks after TAC operation compared to wild-type animals. In isolated neonatal rat ventricular cardiac myocytes stimulation with angiotensin II and phenylephrine enhanced GRK2 expression leading to enhanced signaling via protein kinase B (PKB or Akt), consecutively inhibiting glycogen synthase kinase 3 beta (GSK3β), such promoting nuclear accumulation and activation of nuclear factor of activated T-cells (NFAT). Cardiac myocyte hypertrophy induced by in vitro GRK2 overexpression increased the cytosolic interaction of GRK2 and phosphoinositide 3-kinase γ (PI3Kγ). Moreover, inhibition of PI3Kγ as well as GRK2 knock down prevented Akt activation resulting in halted NFAT activity and reduced cardiac myocyte hypertrophy. Our data show that enhanced GRK2 expression triggers cardiac hypertrophy by GRK2-PI3Kγ mediated Akt phosphorylation and subsequent inactivation of GSK3β, resulting in enhanced NFAT activity.

## Introduction

Cardiac hypertrophy is an initially adaptive mechanism aimed at maintaining cardiac output in response to increased biomechanical stress such as pressure overload in arterial hypertension or aortic stenosis. Cardiac hypertrophy is characterized by reactivation of the fetal gene program, accelerated protein synthesis and adaption of sarcomere structure causing increased cardiac myocyte size. As such, cardiac hypertrophy is temporarily able to normalize wall tension but on long term may increase mortality and promote heart failure (HF) [1, 2].

In clinical conditions leading to cardiac hypertrophy and HF, β-adrenergic receptor (β-AR) blockers, inhibitors of the angiotensin converting enzyme, and mineralocorticoid receptor antagonists are guideline recommended standard therapies, protecting the heart from increased neurohumoral stimulation [3]. The fact that disease progression can be ameliorated by these approaches, despite chronically increased wall stress, indicates the critical role of the sympathetic nervous system (SNS) and renin-angiotensin-aldosterone system (RAAS) in the pathophysiology of cardiac hypertrophy and HF. As these neurohumoral systems mainly transmit their signals via G-protein coupled receptors (GPCRs), understanding of the GPCR dependent signaling likely reveals novel therapeutic approaches.

The cytosolic serin/threonine kinase G-protein coupled receptor kinase 2 (GRK2) represents a critical regulator of cardiac GPCR signaling, participating in HF development and progression [4, 5]. Via binding of the Gβγ-subunit of dissociated G-proteins GRK2 transfers to the sarcolemma specifically phosphorylating agonist-activated GPCRs [5]. Phosphorylation facilitates β-arrestin binding, displacement of the associated G-proteins and uncoupling of the receptor from its downstream targets [6]. Subsequently β-arrestins promote clathrin-mediated receptor endocytosis and degradation, thus reducing neurohumoral responsiveness, which leads to further rise of SNS and RAAS activity [7]. Recently, various additional non GPCR effects of GRK2 in HF have been discovered. These include promotion of insulin resistance [8], alterations of L-type calcium channel Ca2+ handling [9] as well as pro apoptotic effects on mitochondria [10].

Both, human HF and animal models of cardiac stress such as myocardial infarction or genetic cardiomyopathies present upregulation of GRK2 protein expression associated with cardiac hypertrophy [4, 11, 12]. We could previously demonstrate that GRK2 inhibition by genetic ablation or exposure to an inhibitory peptide significantly improves cardiac function and survival in various HF models [4, 11, 13, 14]. Furthermore, reductions of heart-weight to body-weight ratio, inhibition of adverse cardiac remodeling as well as attenuated fetal gene induction following GRK2 ablation or inhibition were commonly observed.

Reduced adverse remodeling after reduction of GRK2 levels or activity could result from improved contractility or a critical role of this kinase in cardiac hypertrophy signaling pathways. As GRK2 connects GPCRs to a multitude of downstream effectors, we hypothesized that GRK2 modulates signaling pathways linked to cardiac hypertrophy.

For the first time we could demonstrate that GRK2 participates in cardiac hypertrophy by nuclear NFAT activation. Analysis of conditional GRK2 knockout mice (GRK2KO) revealed decreased hypertrophy following transverse aortic constriction (TAC). *In vitro* we found the protein kinase B (PKB/Akt)/ glycogen synthase kinase 3 beta (GSK3β) pathway activated when GRK2 expression is increased, resulting in nuclear NFAT accumulation. Finally we could confirm GRK2 dependent NFAT activation in our TAC *in vivo* model and this activation could be abolished by GRK2 knockout.

## Methods

An expanded Materials and Methods section is available in the Online Data Supplement (S1 File).

### Experimental animals

All animal procedures and experiments were carried out strictly according to National Institutes of Health Guidelines on the Use of Laboratory Animals and have been prospectively approved by the Animal Care and Use Committee of Thomas Jefferson University. For surgery Isoflurane anesthesia was used. Tribromoethanol (Avertin) was used as an anesthetic for echocardiography as well as for euthanasia. All efforts were made to minimize animal suffering. Conditional mice bearing floxed GRK2 alleles were described previously [4, 15]. GRK2KO (alpha myosin heavy chain-Cre-recombinase/GRK2flox/flox, αMHC-Cre/GRK2flox/flox) and wild-type (WT) (GRK2flox/flox) mice were maintained on a C57BL/6 genetic background. Male GRK2KO and WT mice were 8–14 weeks of age when entering the study. Unstressed normal mice and mice with TAC were studied. At study end hearts were excised, weighed and snap frozen in liquid nitrogen.

### TAC operation

TAC operation was performed as described previously [16]. For a detailed description see supplemental methods.

### Echocardiography and Doppler measurement

Transthoracic echocardiography was performed as previously described [4]. Doppler measurement of the systolic pressure gradient (SPG) across the TAC site was performed in sedated animals 2 days after TAC.

### Isolation of mouse ventricular myocytes

Adult mouse cardiac myocytes were isolated from sham and TAC operated WT and GRK2KO mice as previously described [4, 17].

### Cell culture

Primary cultures of neonatal rat ventricular cardiac myocytes (NRVMs) were prepared from 1–2 days old Wistar rats as described previously [18].

NRVMs were stimulated either with 10^−7^ M Angiotensin II (ANG II, Merck-Millipore, Darmstadt, Germany) or 10^−4^ M Phenylephrine (PE, Sigma Aldrich, St. Louis, MO, USA) to induce cellular hypertrophy.

### GRK2 knockdown using siRNA

siRNA molecules were designed and delivered by Ambion (Ambion, Berlin, Germany). Using High Perfect transfection reagents (Qiagen, Venia, Netherlands) siGRK2 (sense *5’ UCA AGU UAC UGG ACA GUG A 3’*, anti-sense *3’ UCA CUG UCC AGU AAC UUG A 5’*) and a non-silencing control (sense *5‘ AGC AUU CAU UCG CGU UGG 3‘*, anti-sense *3‘ CCA ACG CGA AUG AAU GCU 5‘*) were transfected into NVRMs 48h and again 96h after isolation. Transfected cells were recovered for 24h and treated with prohypertrophic stimulants (PE, ANG II) for 24h for RT-PCR analysis or 48h for protein analysis. Knockdown of GRK2 was confirmed by Western blotting.

### Kinase inhibition

For pharmacological inhibition of PI3Kγ, Wortmannin (Sigma Aldrich, St. Louis, MO, USA) was added to the respective well to reach a final concentration of 0.1 μM. For inhibition of Akt, MK-2206 (Santa Cruz, Dallas, TX, USA) was added to the respective well to reach a final concentration of 3 μM.

### Western blotting

Western blots were performed as previously described [19]. The following antibodies were used for immunoblotting (IB): GRK2 (sc-562, Santa Cruz, Dallas, TX, USA), GSK3β (sc-9166, Santa Cruz), phospho-GSK3β (sc-373800, Santa Cruz), Akt (#9272, Cell Signaling, Danvers, MA, USA), phospho-AKT (Ser473) (#4051, Cell Signaling), phospho-AKT (Thr308) (#2965, Cell Signaling), GAPDH (Merck-Millipore, Billerica, MA, USA).

### Immunofluorescence

The cells were fixed in 3% paraformaldehyde (PFA) and permeabilized using 0.05% Triton-X100 buffer. For actin staining, cells were stained with a monoclonal antibody against α-actinin (1:500, 4°C, Sigma-Aldrich, St. Louis, MO, USA) and mounted in Fluoromount G (Biozol, Eching, Germany). DAPI (4',6-diamidino-2-phenylindol, Invitrogen, Carlsbad, CA, USA) was used for nuclear counterstain. Images were acquired using an Olympus IX81 florescence microscope (Olympus, Hamburg, Germany). Surface area was quantified by capturing the complete boundary using Sigma Scan software (Aspire Software international, Ashborn, Virginia, USA).

### Proximity ligation assay

The Duolink-PLA assay (Olink, Uppsala, Sweden) was performed according to manufacturer’s protocol with minor modifications regarding antibody concentration. Antibodies for NFATc4 (sc-13036, Santa Cruz, Dallas, TX, USA), NFATc1 (sc-1149, Santa Cruz), GRK2 (sc-18409, Santa Cruz) and PI3Kγ (sc-7177, Santa Cruz) were added at a concentration of 6.7 μg/ml. The green fluorescence dots of each ligation, indicating protein-protein interaction, were detected using a Nikon laser scanning confocal microscope (Nikon C2+, Düsseldorf, Germany) and related to cell area. Other fluorescence assays were analyzed using an Olympus IX81 florescence microscope (Olympus, Hamburg, Germany). Fluorescence microscope images and intensity signals (green spots) were counted using ImageJ software (National Institute of Health, Bethesda, MD, USA).

### Luciferase assay

Activity of NFAT was determined by transducing cardiac myocytes with a luciferase reporter linked to a promoter with three NFAT sites (gift from Prof Dr. Johannes Backs, University of Heidelberg, Germany). NRVMs were first infected with recombinant adenovirus harboring the luciferase reporter construct for 24 h in 0.5% serum-reduced culture medium before stimulation with ANG II. Cells were lysed 24 h after stimulation. After centrifugation (14,000 rpm for 15 min) 200 μl luciferase substrate (E1500, Promega, Madison, WI, USA) were added to the cell lysate and immediately analyzed on a luminometer (Berthold, Bad Wildbad, Germany) according to the manufacturer’s specifications. Relative light units were normalized to total protein concentration.

### RNA Isolation and quantitative real-time polymerase chain reaction (RT-PCR)

Quantitative real-time polymerase chain reaction was performed as previously described [4].

### Statistics

All data in the text and figures are presented as mean ± standard error of the mean (SEM). Statistical significance was analyzed using one-way or two-way ANOVA followed by Tukey’s or Bonferroni’s post-hoc test for multiple comparisons if appropriate. Statistical significance between two groups was determined using the two-tailed Student’s t-test or Mann-Whitney-U-test if appropriate. Data analysis was performed using Prism 6 (GraphPad, La Jolla, CA, USA). A P-value <0.05 was considered to be statistically significant.

## Results

### Cardiac-specific GRK2KO prevents pressure overload induced cardiac hypertrophy *in vivo*

**Fig 1:**
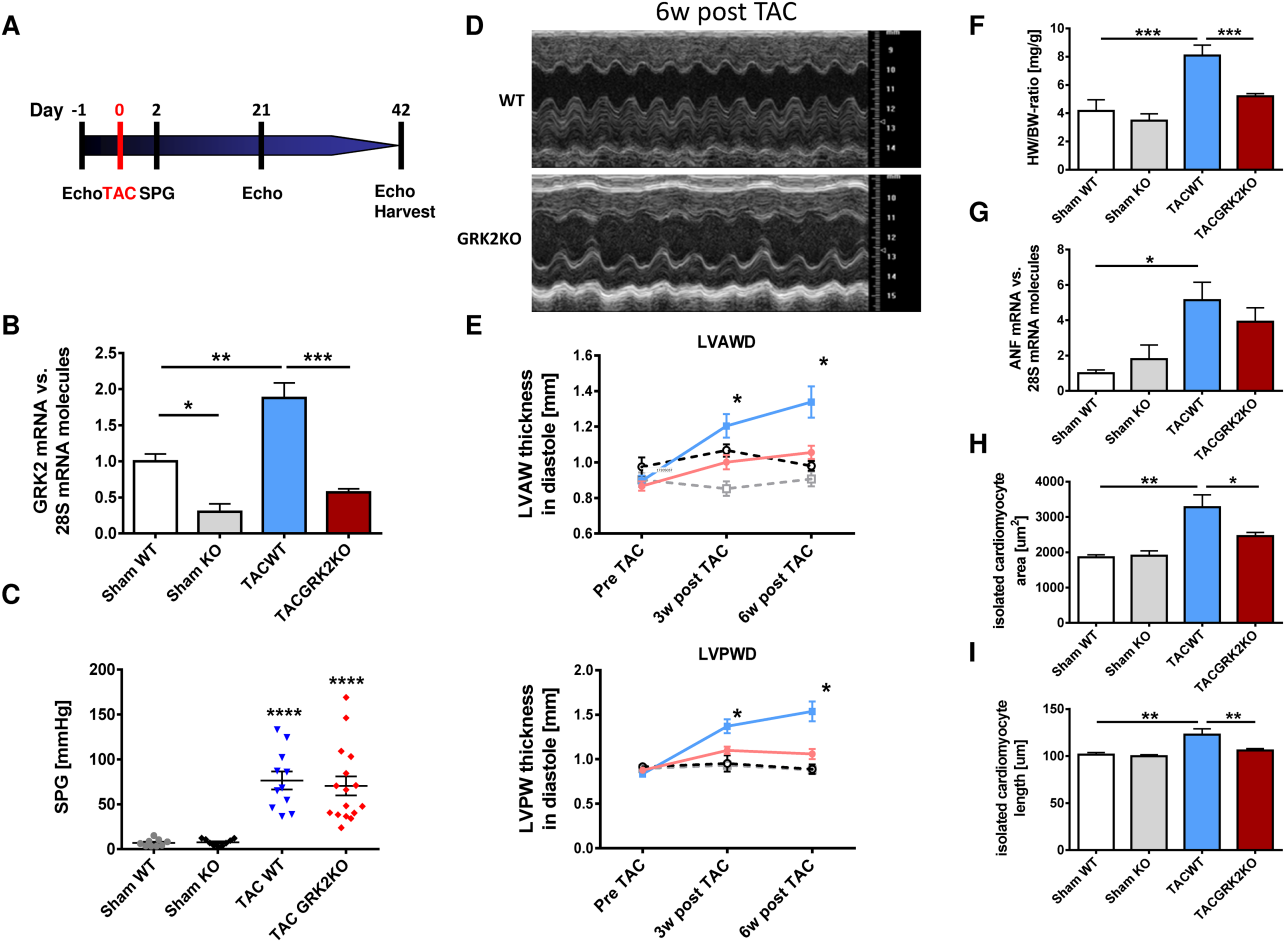
G-protein coupled receptor kinase 2 knockout (GRK2KO) attenuates cardiac hypertrophy in vivo. (A) Study design and *in vivo* model of pressure overload hypertrophy. Experimental protocol, echocardiography (Echo), transverse aortic constriction (TAC), Doppler measurement of systolic pressure gradient (SPG), organ harvest at study end (Harvest). (B) GRK2 mRNA expression normalized to 28s RNA 6 weeks after TAC operation, n = 3 for KO, n = 4–5 for WT groups, * P < 0.05, ** P < 0.01, *** P < 0.001. (C) Doppler measurement of aortic systolic pressure gradient (SPG) after TAC, n = 9 for sham, n = 11 for TAC WT and n = 16 for TAC GRK2 group. *** P < 0.0001 for TAC vs. Sham groups. (D) Representative M-Mode echocardiography recordings at study end, 6 weeks post TAC. (E) Echocardiographic assessment of ventricular remodeling. Left ventricular anterior/posterior wall thickness/diameter (LVAWD and LVPWD) determined before, 3 weeks and 6 weeks after TAC or sham operation in GRK2KO and wild-type (WT) animals. TAC GRK2 KO (red line, n = 18), TAC WT (blue line, n = 13), Sham GRK2KO (black dotted line, n = 6), Sham WT (grey dotted line, n = 8), * P < 0.05 TAC WT vs. TAC GRK2KO. (F) Heart-weight to body-weight ratio (HW/BW) measured at study end, 6 weeks after TAC, n = 4 for sham, n = 9 for TAC WT and n = 12 for TAC GRK2 KO group. (G) Atrial natriuretic factor (ANF) mRNA normalized to 28S RNA and Sham WT group showing attenuated ANF upregulation in GRK2 KO animals after TAC, n = 3–6. (H + I) Measurements of cell area and cell length from isolated adult ventricular cardiac myocytes (AVCM), n = 4 for sham groups, n = 9 for TAC WT and n = 12 for TAC GRK2 K, 20 cells per animal were measured; F-I * P < 0.05; ** P < 0.01; *** P < 0.001.

We have previously described and characterized cardiac myocyte specific conditional GRK2KO mice (αMHC-Cre/GRK2flox/flox) and respective control wild-type (WT) mice (GRK2flox/flox) [4]. Mice of both lines underwent TAC operation for induction of cardiac hypertrophy or sham operation (sham) (Fig 1A). Post TAC a significant increase in GRK2 mRNA levels in WT animals was observed (Fig 1B). In GRK2KO animals GRK2 mRNA levels were reduced at baseline with only mild increases post TAC operation compared to WT animals (Fig 1B). Echocardiographic assessment of the Doppler flow velocity at the aortic constriction site confirmed similar increases in flow velocity and thus pressure gradients between GRK2KO and WT groups after TAC operation (Fig 1C).

WT animals showed significant left ventricular (LV) hypertrophy with increase in LV wall thickness 3 weeks and more pronounced 6 weeks after TAC. Interestingly, in GRK2KO mice adverse remodeling was significantly reduced (Fig 1D and 1E, for further echocardiographic parameters see S1 Fig).

In post mortem analysis GRK2KO mice revealed significantly less LV hypertrophy represented by reduced heart-weight to body-weight ratio (Fig 1F) and reduced dimensions of isolated cardiac myocytes (cell area and cell length) compared to WT mice after TAC (Fig 1H and 1I). Fetal gene expression was significantly elevated only in WT mice post TAC (Fig 1G).

### GPCR stimulation with ANG II and PE induce myocardial hypertrophy and GRK2 upregulation *in vitro*

**Fig 2:**
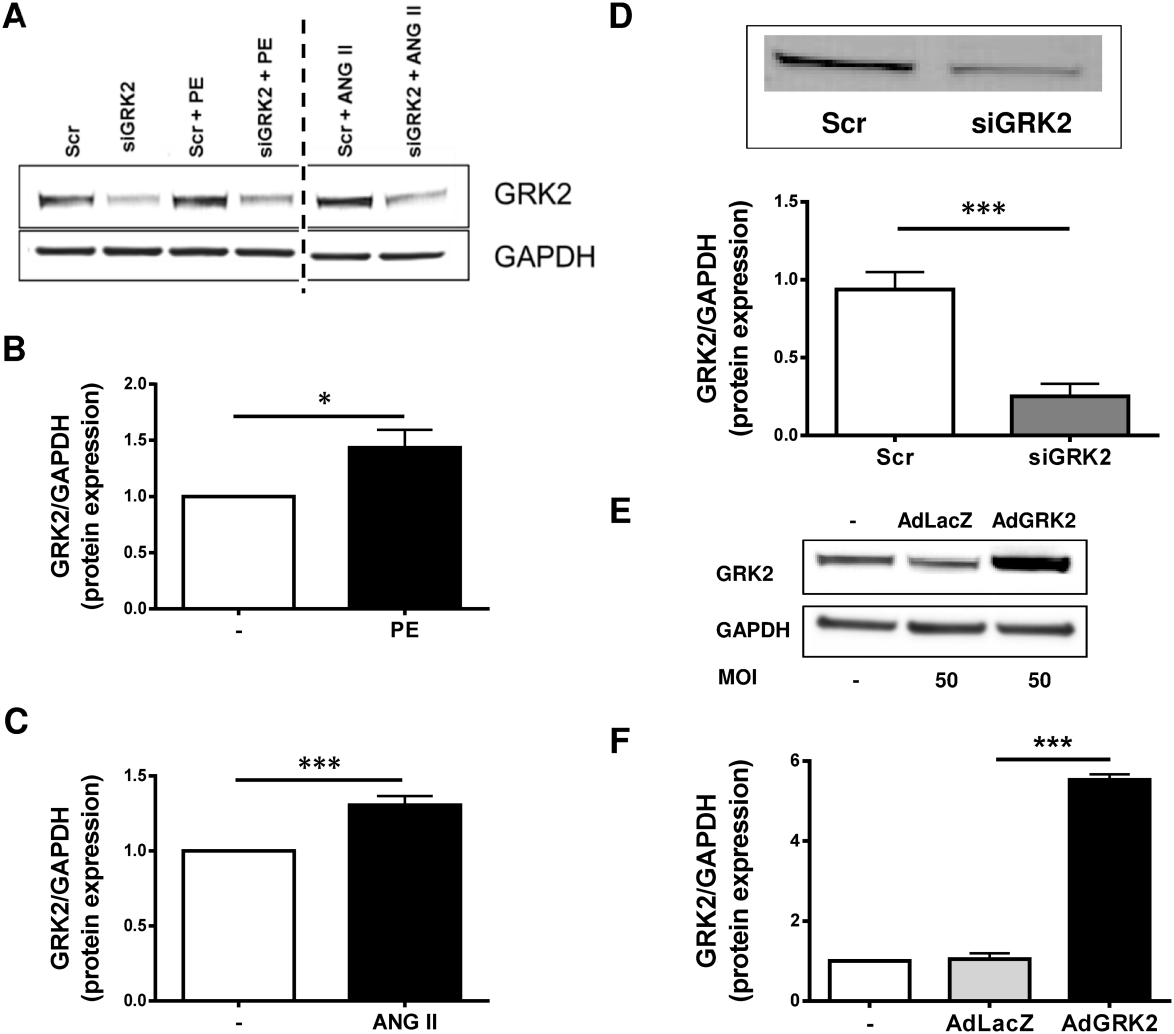
G-protein coupled receptor kinase 2 (GRK2) expression is regulated by G-protein coupled receptor (GPCR) stimulation *in vitro*. (A) representative Western Blots of GRK2 expression in neonatal rat ventricular myocytes (NRVMs) after stimulation with Phenylephrine (PE), or Angiotensin II (ANG II), GAPDH as loading control. (B) Quantification of GRK2 protein expression after PE stimulation normalized to GAPDH and unstimulated (-) NRVM as control. Samples shown on each lane are blotted, antibody stained and developed on the same western blot membrane. Vertical dotted line indicates 2 excluded samples with non ANG II/PE GPCR stimulation. n = 10, * P < 0.05. (C) Quantification of GRK2 protein expression after ANG II stimulation normalized to GAPDH, n = 11, *** P < 0.001. (D) GRK2 knockdown by siRNA. Treatment of NRVM with siRNA against GRK2 (siGRK2, n = 6) and scrambled siRNA as control (Scr, n = 4), ** P < 0.01. (E) Adenoviral GRK2 overexpression. Representative Western Blots of GRK2 protein expression in untreated NRVMS, NRVMs treated with a control adenovirus harboring β-galactosidase (AdLacZ) and NRVMs after treatment with an adenovirus expressing GRK2 (AdGRK2), MOI = multiplicity of infection. (F) Quantification of GRK2 protein expression following AdLacZ or AdGRK2 transfection normalized to GAPDH as loading control and normalized to untreated cells (-), n = 2.

To further define the molecular role of GRK2 in cardiac hypertrophy, an *in vitro* model was established. Therefore, isolated NRVMs were stimulated with either ANG II or PE. Both, ANG II and PE exposure resulted in significant increase in GRK2 protein expression comparable to our *in vivo* findings (Fig 2A–2C). GRK2 expression levels were modulated by siRNA mediated knockdown after ANG II or PE treatment (siGRK2, Fig 2A and 2D) or adenoviral GRK2 overexpression (AdGRK2, Fig 2E and 2F).

### GRK2 knock down inhibits ANG II and PE mediated hypertrophy *in vitro*

**Fig 3:**
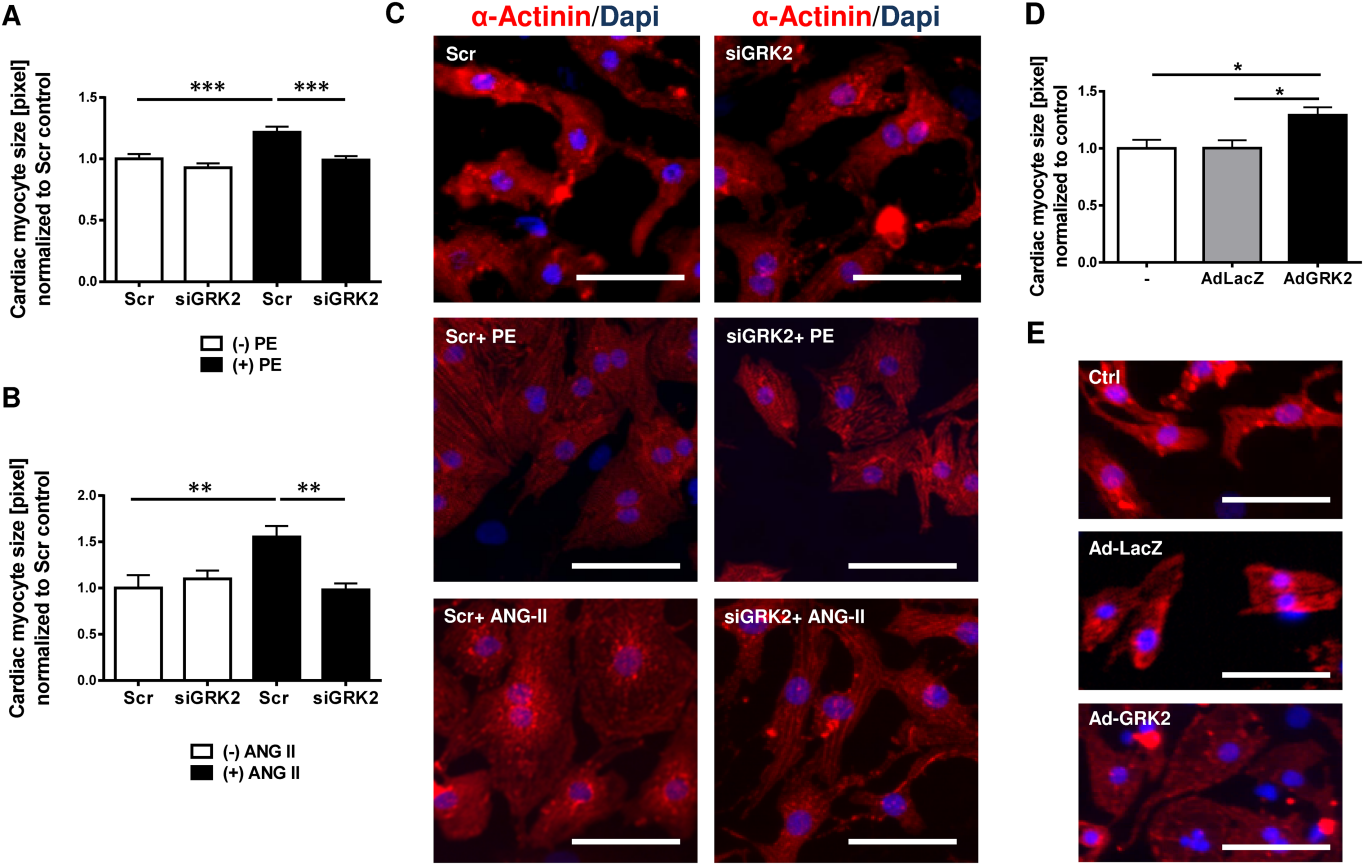
G-protein coupled receptor kinase 2 (GRK2) promotes cardiac myocyte hypertrophy. (A-C) GRK2 knockdown by siGRK2 attenuates G-protein coupled receptor (GPCR) induced cardiac hypertrophy. (A) Quantification of cardiac myocyte cell size from immunofluorescence stains of neonatal rat ventricular myocytes (NRVMs) following siRNA mediated GRK2 knockdown and GPCR stimulation by phenylephrine (PE), >200 NRVM per condition from 3 independent cell preparations were analyzed, *** P < 0.001. (B) Quantification of cardiac myocyte cell size following siRNA mediated GRK2 knockdown and Angiotensin II (ANG II) treatment, >100 NRVM per condition from 3 independent cell preparations were analyzed, **, P < 0.01. (C) Representative immunofluorescence images of NRVMs under respective conditions. α-Actinin is stained red for demarcation of cell dimensions, blue DAPI staining marks nuclei. (D, E) GRK2 overexpression promotes hypertrophy. (D) Quantification of cardiac myocyte cell size from immunfluorescence stains of NRVMs following treatment with an adenovirus harboring GRK2 (AdGRK2) or β-galactosidase/LacZ as control (AdLacZ), >100 NRVM per condition from 3 independent cell preparations were analyzed, *, P < 0.05. (E) Representative images of immunofluorescence of NRVMs (DAPI and α-Actinin) following AdGRK2 or AdLacZ transduction compared or untreated (-) cells. Scale bar (C and E): 50 μm.

Stimulation of NRVM either using ANG II or PE resulted in significant increase in cardiac myocyte size compared to unstimulated cells (Fig 3A–3C). Interestingly, GRK2 knockdown using siRNA against GRK2 prevented myocyte hypertrophy after ANG II and PE stimulation. Cell size in unstimulated cells was not altered, suggesting that increased GRK2 expression levels are required for the prohypertrophic effects of GPCRs (Fig 3A–3C). Adenoviral mediated GRK2 overexpression (AdGRK2) led to a significant increase of cardiac myocyte cell size compared to NRVMs treated with a control vector (adenovirus expressing LacZ, AdLacZ) (Fig 3D and 3E). mRNA upregulation of fetal genes such as atrial natriuretic factor (ANF), brain natriuretic peptide (BNP) and β-myosin heavy chain (βMHC) due to ANG II and PE stimulation was reduced by GRK2 siRNA knockdown (S2 Fig).

### GRK2 promotes nuclear NFAT accumulation

**Fig 4:**
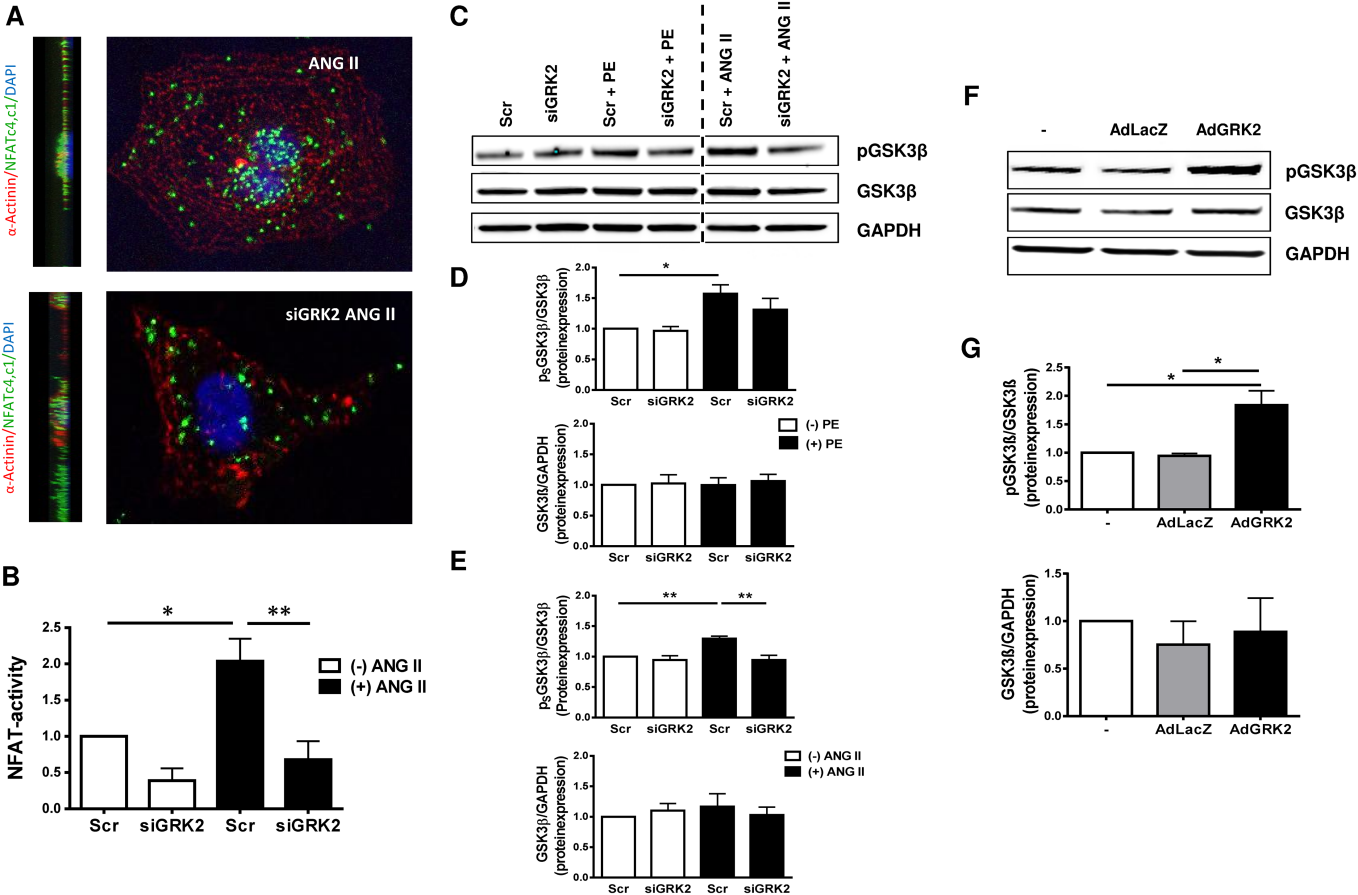
G-protein coupled receptor kinase 2 (GRK2) regulates nuclear factor of activated T-cells (NFAT) nuclear activity via glycogen synthase kinase 3β (GSK3β). (A, B) G-protein coupled receptor (GPCR) induced nuclear NFAT activity is decreased by GRK2 knockdown. (A) Representative immunofluorescence images of neonatal rat ventricular cardiac myocytes (NRVMs) under respective treatment conditions; NFAT subunits are marked using a proximity ligation assay (respective antibodies against each NFAT c1 and c4) resulting in green fluorescence, α-Actinin (red), DAPI (blue). (B) Transcriptional NFAT activity was evaluated using the NFAT-luciferase-reporter assay system, n = 3–4, * P< 0.05; ** P < 0.01. (C-G) GPCR stimulation results in increased GSK3β phosphorylation mediated by GRK2. (C) Representative Western Blots of GSK3β and phosphorylated GSK3β (pGSK3β) under respective treatment conditions, GAPDH served as loading control. Samples shown on each lane are blotted, antibody stained and developed on the same western blot membrane. Vertical dotted line indicates 2 excluded samples with non ANG II/PE GPCR stimulation. Quantification of GSK3β phosphorylation and total GSK3β protein under PE (D, n = 4) and ANG II (E; n = 6) stimulation, all values normalized to scrambled control (Scr), * P< 0.05, ** P < 0.01. (F, G) GRK2 overexpression promotes GSK3β phosphorylation. (F) Representative Western Blots of pGSK3β and GSK3β after transfection with an adenovirus harboring GRK2 (AdGRK2) or β-galactosidase/LacZ as control (AdLacZ) or untreated cells (-), GAPDH served as loading control. (G) Quantification of pGSK3β and total GSK3β with and without GRK2 overexpression, n = 3, * P < 0.05.

NFAT is an important transcription factor promoting cardiac hypertrophy. NFAT activity is modulated by GSK3β and calcineurin by phosphorylation or dephosphorylation of serine/threonine residues in the N-terminal regulatory regions of the NFAT protein, thus regulating NFAT-transfer between the nuclear and cytosolic compartment [20]. Phosphorylation masks the NFAT nuclear import sequence resulting in nuclear export and inactivation of transcriptional activity. Using a NFAT-Luciferase assay we could successfully demonstrate ANG II dependent nuclear accumulation of NFAT. Interestingly, siRNA mediated GRK2 knockdown significantly reduced nuclear NFAT activity upon ANG II stimulation (Fig 4B). By a proximity ligation assay under ANG II stimulation we observed a similar reduction of nuclear NFAT localization by siGRK2 treatment (Fig 4A).

### GRK2 mediates pro hypertrophic signaling via Akt and GSK3β

**Fig 5:**
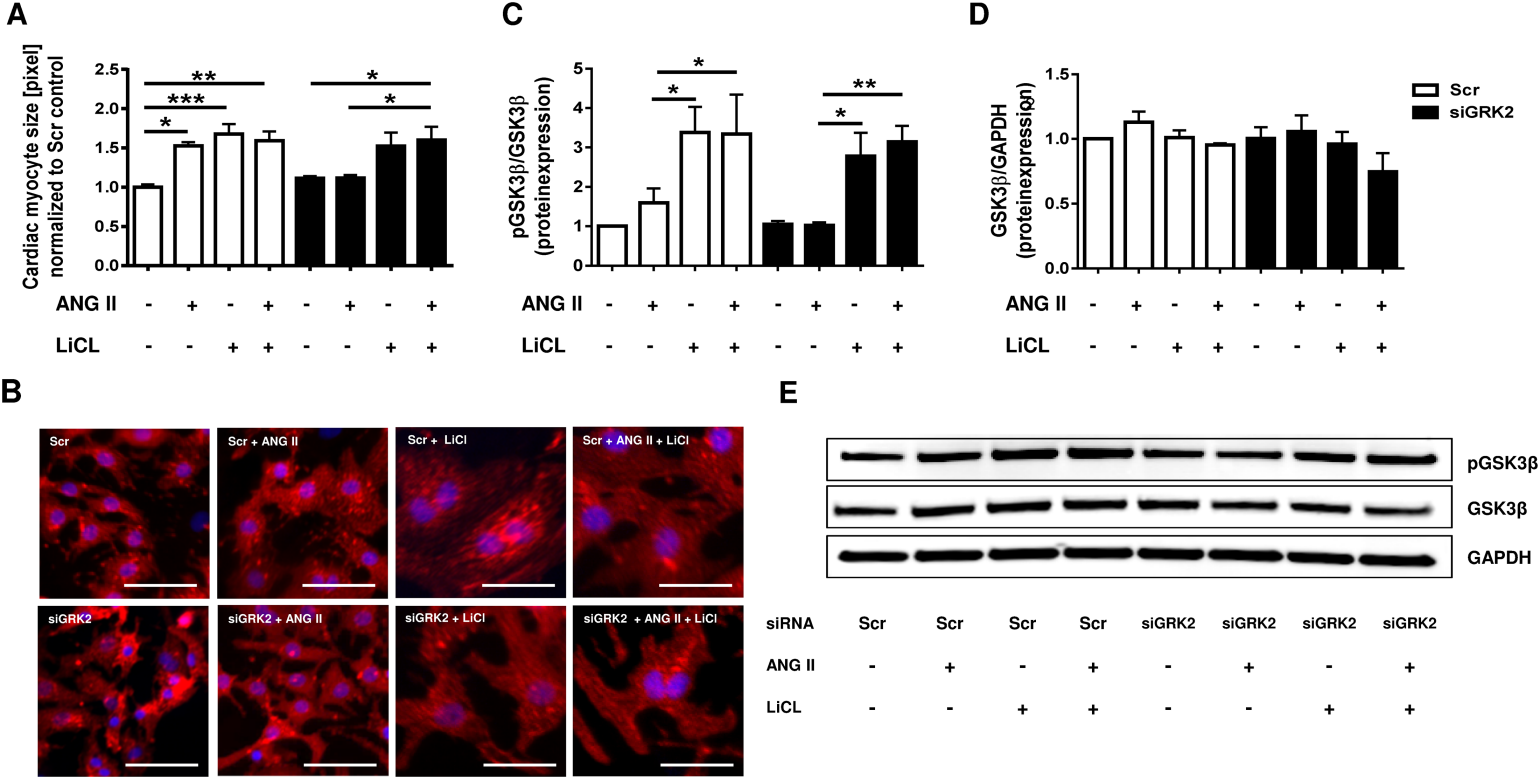
Anti-hypertrophic effects of GRK2 knock down can be abolished by GSK3β inhibition. (A) Quantification of cardiac myocyte cell size and (B) representative immunofluorescence stains of neonatal rat ventricular myocytes (NRVMs) with or without siRNA mediated GRK2 knockdown, GPCR stimulation by ANGII and/or GSK3β inhibition by lithium chloride (LiCl). Quantification of (C) phosphorylated GSK3β to total-GSK3β protein expression (n = 6–12) and (D) GSK3β to GAPDH (n = 5) including (E) representative Western Blots under respective treatment conditions, all values normalized to scrambled control (Scr). >200 NRVM per condition from 3 independent cell preparations were analyzed for cell size determination, * P < 0.05, ** P < 0.01, *** P < 0.001. Scale bar (B): 50 μm.

The serine/threonine kinase GSK3β, a regulator of NFAT activity, is controlled by phosphorylation of the serine 9 residue by Akt. An increase in phosphorylation results in inactivation of GSK3β kinase activity. Western Blot analysis of GSK3β phosphorylation status revealed significant increase in GSK3β phosphorylation under PE and ANG II stimulation, while GSK3β protein expression remained unaffected (Fig 4C–4E). In contrast, siGRK2 co-treatment led to an insignificant decrease in GSK3β phosphorylation under PE stimulation (Fig 4C and 4D) and could even significantly reduce GSK3β phosphorylation under ANG II stimulation (Fig 4C and 4E). GRK2 overexpression by AdGRK2 enhanced GSK3β phosphorylation compared to untreated or control AdLacZ treated cells (Fig 4F and 4G). Inhibition of GSK3β by the selective GSK3 inhibitor lithium chloride (LiCl) resulted in marked increase in cardiomyocyte cell size, similar to ANG II stimulation (Fig 5A and 5B). While siGRK2 treatment abolished ANGII induced hypertrophy under basal conditions, additional LiCl exposure reinitiated hypertrophy of NRVMs. Correspondingly, LiCl exposure results in excessive GSK3β phosphorylation independent of siGRK2 treatment, suggesting GSK3 β as central mediator of GRK2 dependent cardiomyocyte hypertrophy (Fig 5C–5E).

**Fig 6:**
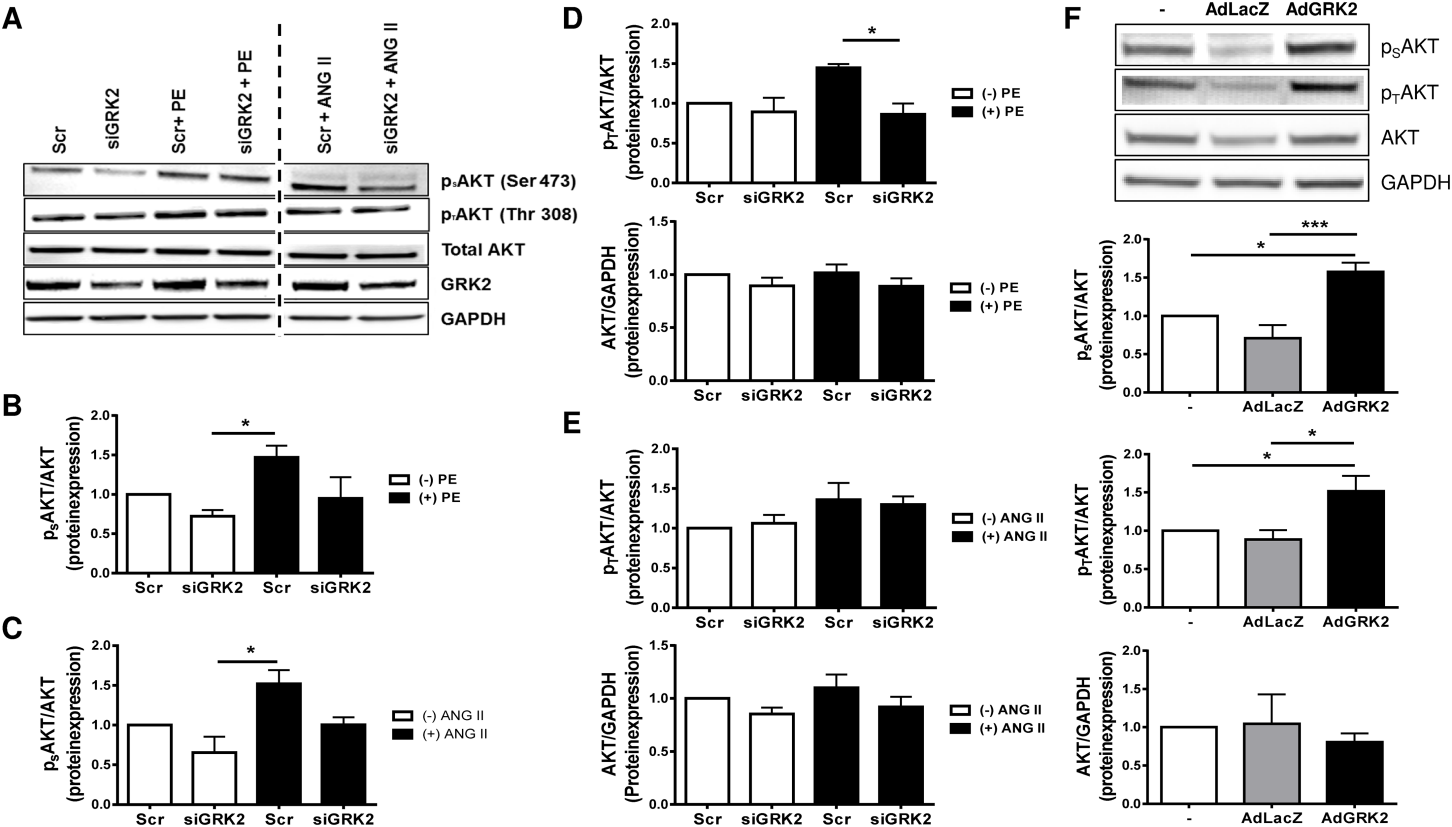
G-protein coupled receptor kinase 2 (GRK2) regulates proteinkinase B (Akt) phosphorylation. (A-E) G-protein coupled receptor (GPCR) promoted Akt phosphorylation depends on GRK2. (A) Representative Western Blots of Akt phosphorylation at residues Ser 473 (p_S_AKT) and Thr 308 (p_T_AKT), total Akt expression, GRK2 expression and GAPDH under respective conditions. Samples shown on each lane are blotted, antibody stained and developed on the same western blot membrane. Vertical dotted line indicates 2 excluded samples with non ANG II/PE GPCR stimulation. Quantification of p_S_AKT under PE (B; n = 4) and ANG II (C; n = 3) and p_T_AKT under PE (D; n = 3) and ANG II (E; n = 4) stimulation and total Akt under respective treatment conditions, values normalized to control scrambled siRNA (Scr), * P < 0.05. (F) GRK2 overexpression promotes full Akt phosphorylation. Representative Western Blots and quantification of p_S_AKT (n = 7), p_T_AKT (n = 7) and total Akt (n = 4) expression after transfection with an adenovirus harboring GRK2 (AdGRK2) or β-galactosidase/LacZ as control (AdLacZ), GAPDH served as loading control. Values normalized to untreated control (-). * P < 0.05, *** P < 0.001.

As Akt is known to be a critical regulator of GSK3β [21] we analyzed Akt phosphorylation as well as Akt protein levels. Akt has two main regulatory phosphorylation sites [22], namely Serine 473 (Ser473) and Threonin 308 (Thr308). Thr308 phosphorylation is increased following stimulation with PE, while siRNA mediated GRK2 knockdown led to a significant reduction (Fig 6A and 6D). In contrast, ANG II stimulation resulted only in a moderate increase of Thr308 phosphorylation (Fig 6A and 6E). PE and ANG II stimulation increased phosphorylation on the Akt Ser473 site, which was reduced by siRNA knockdown of GRK2 (Fig 6A–6C). Under both stimulants total Akt protein level remained unchanged compared to control. Adenoviral overexpression of GRK2 resulted in significant increase in Akt phosphorylation both on Thr308 as well as on Ser473, supporting the notion that full Akt activation is closely linked to the GRK2 expression level (Fig 6F).

### Inhibition of PI3Kγ and Akt attenuates GRK2 dependent cardiac hypertrophy and NFAT activation

**Fig 7:**
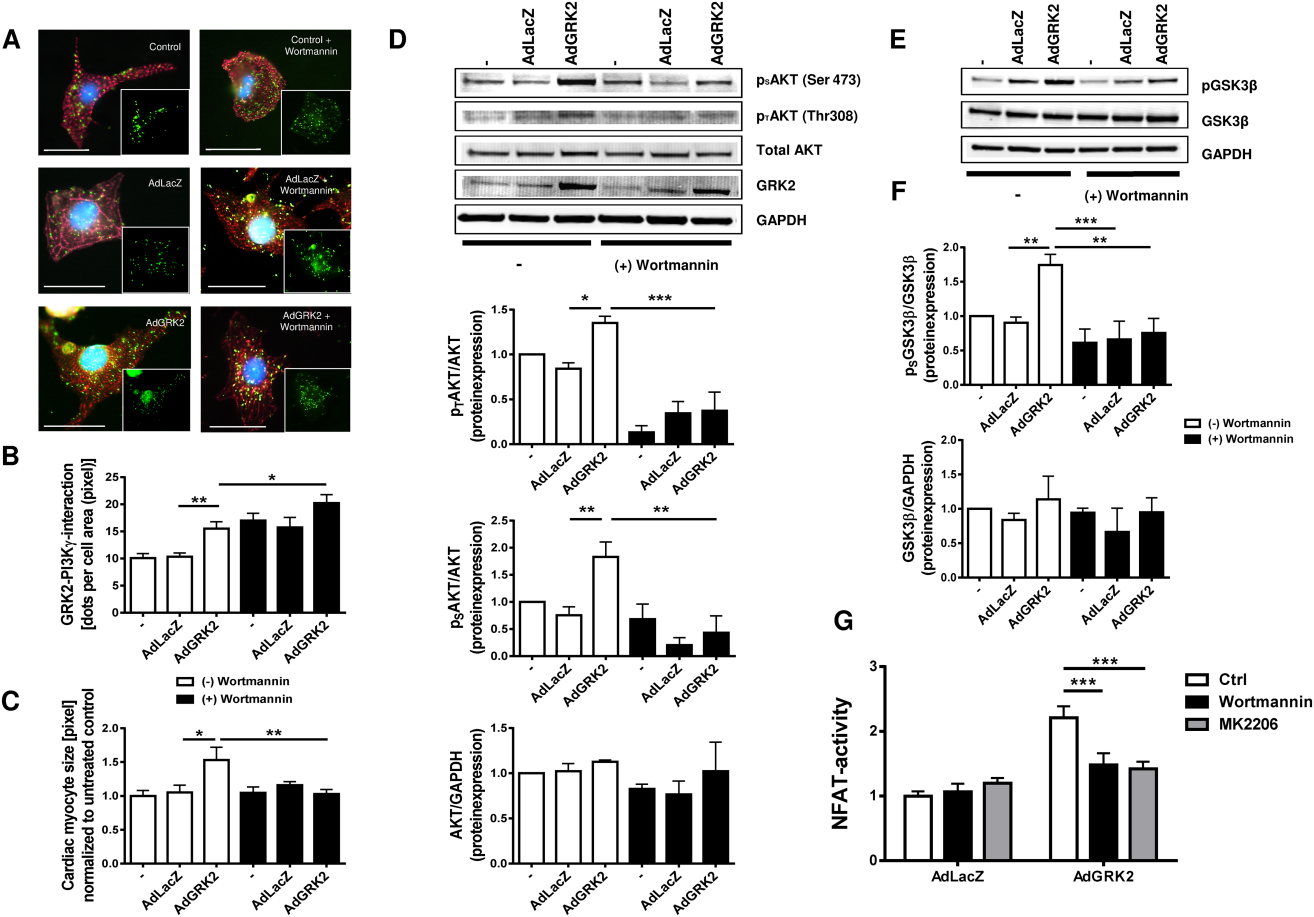
Interaction of G-protein coupled receptor kinase 2 (GRK2) and phosphoinositide 3-kinase γ (PI3Kγ) mediates cardiac hypertrophy and NFAT activation. (A-C) GRK2 and PI3Kγ interaction promotes cardiac hypertrophy. (A) Representative immunofluorescence images and (B) quantification of GRK2-PI3Kγ-interaction by proximity ligation assay and quantification of cardiac myocyte size (C) in cells with and without pharmacological PI3Kγ inhibition by Wortmannin; for modulation of GRK2 levels neonatal rat ventricular myocytes (NRVMS) were transduced with an adenovirus harboring GRK2 (AdGRK2) or β-galactosidase/LacZ (AdLacZ) or were not treated with an adenovirus (control); GRK2-PI3Kγ-interaction is visualized by proximity ligation assay were each green dot represents GRK2 and PI3Kγ alignment (green), α-Actinin (red), DAPI (blue), 18–34 individual cells from 2 independent cell preparations were analyzed, * P < 0.05, ** P < 0.01. Scale bar (A): 20 μm. (D) GRK2-PI3Kγ-interaction promotes proteinkinase B (Akt) phosphorylation. Representative Western Blots of Akt phosphorylation at residues Ser 473 (p_S_AKT) and Thr 308 (p_T_AKT), total Akt expression, GRK2 expression and GAPDH under respective conditions, Akt protein expression normalized to untreated (-) cells, n = 3–8, * P < 0.05, ** P < 0.01, *** P < 0.001. (E, F) GRK2-PI3Kγ-interaction promotes phosphorylation of glycogen synthase kinase 3β (GSK3β). Representative Western Blot (E) and quantification (F) of pGSK3β to total GSK3β (n = 4–8) and GSK3β to GAPDH (n = 2–3), values normalized to untreated control (-), ** P < 0.01, *** P < 0.001. (G) PI3Kγ inhibition (by Wortmannin) and Akt inhibition (by MK2206) abrogate NFAT activation induced by adenoviral GRK2 overexpression. (B) Transcriptional NFAT activity was evaluated using the NFAT-luciferase-reporter assay system. NRVM were transduced with an adenovirus harboring luciferase (AdLuc, MOI 25) plus either an adenovirus harboring LacZ (AdLacZ, MOI 25) or GRK2 (AdGRK2, MOI 25) for 48h. Data are normalized to AdLacZ without inhibition, n = 12–18 from 3 different cell preparations, *** P <0.001.

PI3Kγ is a cytosolic serin/threonine kinase which regulates Akt and has been shown to directly interact with GRK2 [23]. We hypothesized that cardiac myocyte hypertrophy due to GRK2 overexpression is PI3Kγ dependent and could be reduced by PI3Kγ inhibition using the pharmacological PI3K-inhibitor Wortmannin. GRK2-PI3Kγ interaction was increased by GRK2 overexpression (Fig 7A and 7B). Inhibition of PI3K further enhanced this interaction, most probably due to local accumulation of inactivated PI3Kγ. Interestingly, while AdGRK2 overexpression resulted in significant increase in cell size, we found robust inhibition of GRK2 induced cardiac myocyte hypertrophy after treatment with Wortmannin (Fig 7C). In line with this, Wortmannin treatment prevented GRK2 induced Akt and GSK3β phosphorylation, suggesting an essential role for GRK2-PI3Kγ interaction in GRK2 mediated cardiac hypertrophy (Fig 7D–7F). Moreover NFAT activation induced by GRK2 overexpression could be attenuated not only by inhibition of PI3Kγ using Wortmannin but also by Akt inhibition by MK2206. This substantiates that the PI3Kγ/Akt axis is the essential signaling pathways of hypertrophy induces by GRK2 overexpression (Fig 7G).

### *In vivo* GRK2KO attenuates expression of the regulator of calcineurin 1 (RCAN)

In order to confirm that NFAT activation is also GRK2 dependently regulated *in vivo*, RCAN expression level in GRK2KO mice following TAC operation were evaluated. Expressionof RCAN, a potent endogenous inhibitor of calcineurin, closely correlates with NFAT activity [24]. Therefore GRK2KO mice and WT control mice were subjected to TAC operation and hearts were collected at either 1 or 4 days following TAC operation.

**Fig 8:**
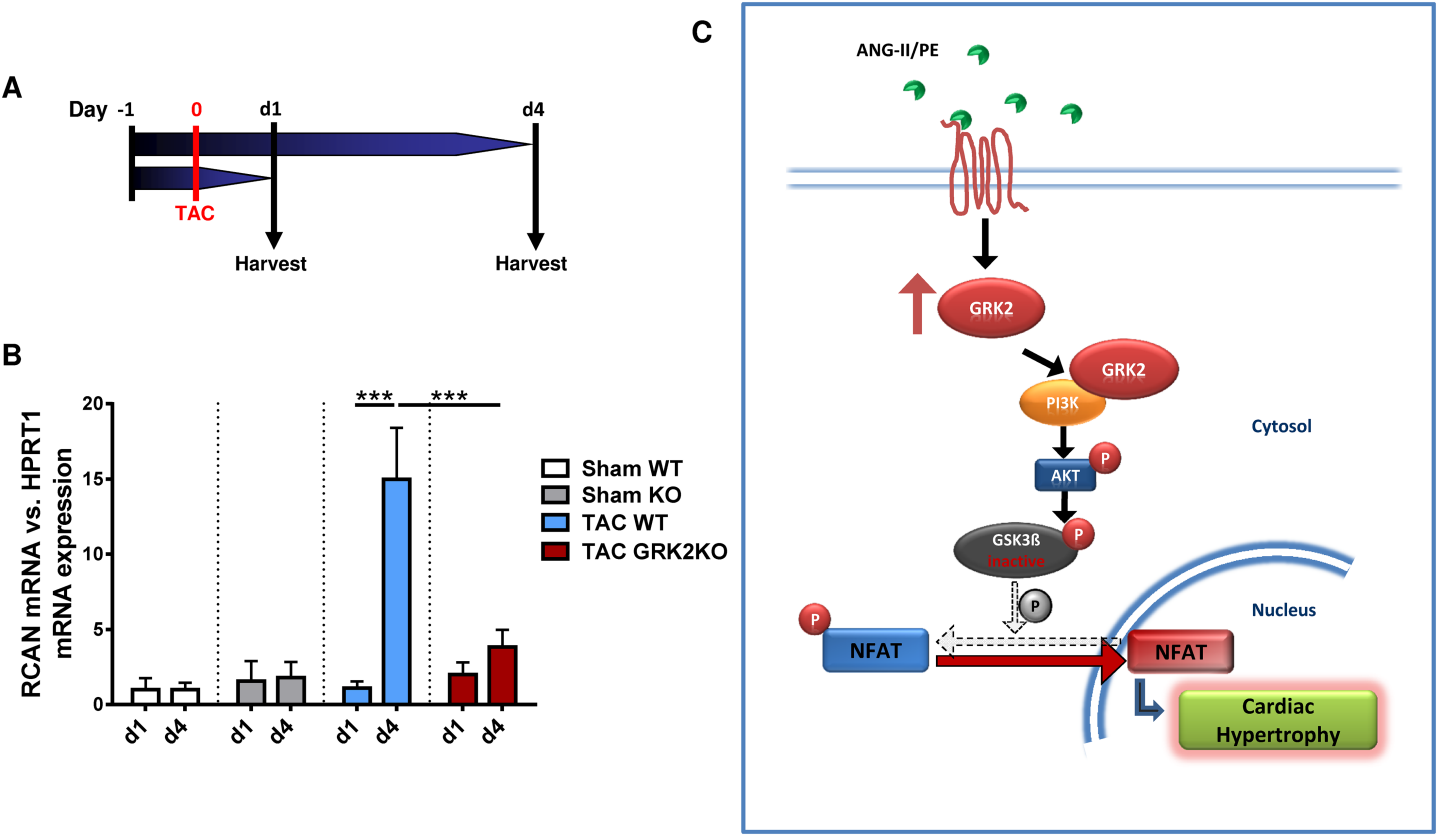
Genetic knockout of GRK2 abolishes GRK2-dependent NFAT activation. (A, B) GRK2 knockout attenuates regulator of calcineurin 1 (RCAN) transcription and thus NFAT activation following transverse aortic constriction. (B) Experimental protocol. Transverse aortic constriction (TAC), organ harvest at study end (Harvest). (C) mRNA expression of NFAT dependent RCAN in GRK2KO mice and respective WT control mice at 1 day and 4 days following TAC operation. n = 4 for sham WT (d1+d4), n = 4 for sham KO (d1+d4), n = 8–10 for TAC WT (d1+d4) and n = 9–10 for TAC GRK2 group (d1+d4). *** P < 0.001. (C) Illustration of proposed signaling pathway for G-protein coupled receptor kinase 2 (GRK2) mediated cardiac hypertrophy. Angiotensin II (ANG II), phenylephrine (PE), phosphoinositide 3-kinase γ (PI3Kγ), protein kinase B (Akt), glycogen synthase kinase 3 β (GSK3β), nuclear factor of activated T-cells (NFAT).

NFAT activity in TAC animals was unaltered at day 1 but significantly increased subsequently. 4 days after TAC surgery mean RCAN mRNA expression was approximately 15x higher compared to sham control animals. Conversely, GRK2KO mice displayed a significantly attenuated RCAN response to pressure overload (Fig 8A+8B).

## Discussion

The role of GRK2 in HF development and progression is well studied, indicating GRK2 as a potential target in the treatment of HF. In this regard, rodent and large animal HF studies could demonstrate beneficial effects of GRK2 inhibition, as both conditional GRK2KO [4] and expression of an inhibitory peptide (βARKct) [13, 14] improved cardiac function. A common finding of GRK2 inhibition was attenuated cardiac remodeling and decreased heart-weight to body-weight ratio in response to cardiac stress [4, 9]. The blunted hypertrophic response might result from overall improved contractility which could indirectly suppress cardiac hypertrophy. However, involvement of GRK2 in GPCR and non-GPCR signaling may also indicate direct modulation of molecular pathways involved in the regulation of cardiac hypertrophy.

In the present study we could demonstrate that enhanced GRK2 expression induced by GPCR stimulation mediates cardiac hypertrophy. *In vivo* pressure overload by TAC significantly increased GRK2 protein expression associated with cardiac remodeling, growth in cardiac myocyte size and volume and activation of the fetal gene program. Cardiac hypertrophy was blunted by conditional GRK2 knockout. *In vitro* PE and ANG II stimulation of NRVMs induced GRK2 expression with consecutive increase in cardiac myocyte cell size. We found nuclear NFAT accumulation to be regulated by GRK2 *in vivo* and *in vitro*. Our data suggest GRK2-PI3Kγ interaction facilitating phosphorylation of downstream targets Akt and GSK3β culminating in nuclear NFAT accumulation (Fig 8C). We therefore propose a novel molecular role for GRK2 in the regulation of cardiac hypertrophy.

### GRK2KO attenuates pressure overload induced cardiac hypertrophy

In order to further characterize GRK2 mediated effects on cardiac hypertrophy signaling, we exposed conditional GRK2KO mice to pressure overload. In WT animals TAC resulted in increases in GRK2 mRNA levels, heart-weight to body-weight ratio, LV wall thickness and cardiac myocyte size constitent with previous reports [25]. Interestingly, GRK2KO animals presented significantly less cardiac hypertrophy with preserved ventricular geometry and only moderate increases in cardiac myocyte size. Similar to our *in vivo* data, Sorriento et al. could ameliorate cardiac hypertrophy in chronically PE stimulated mice by GRK2 knockdown and GRK2 kinase inhibition [26]. We thus conclude that GRK2 promotes cardiac hypertrophy.

### GRK2 regulates cardiac hypertrophy in response to GPCR stimulation

It has been described by Tesmer et al. that GRK2 interacts with Gαq and Gβγ at the plasma membrane [27]. With Gαq promoting cardiac hypertrophy and ANG II and PE being potent stimulators of Gαq via respective GPCRs, we used these agents to model cardiac myocytes hypertrophy in NRVMs. Similar to our *in vivo* findings, *in vitro* GPCR stimulation increased GRK2 expression and promoted cardiac myocyte hypertrophy and fetal gene induction. siRNA knock down of GRK2 reduced cardiac myocyte hypertrophy and attenuated activation of fetal genes. These findings were consistent under both ANG II and PE stimulation. Thus, GRK2 promotes cardiac hypertrophy in response to GPCR stimulation. Notably, the observed prohypertrophic effects are not related to a specific GPCR-subtype but share enhanced GRK2 expression as a common feature facilitating cardiomyocyte hypertrophy. The exact mechanism by which PE and ANG II increase GRK2 expression is not yet fully understood. A complex interplay of distinct signal transduction pathways seems to be able to modulate GRK2 expression [28, 29], including both, GPCR and non-GPCR stimuli [29]. Moreover, proteasomal degradation of GRK2 has been implied as a regulating factor for GRK2 level. Interestingly, the PI3K/Akt activation seems to decrease GRK2 degradation [30] suggesting this as a possible participating mechanism for the increased GRK2 protein level observed in this study.

### GRK2 promotes cardiac hypertrophy via the Akt/PKB-GSK3β-NFAT pathway

We found that GRK2 knockdown diminishes nuclear NFAT accumulation. NFAT is a known transcription factor of the prohypertrophic and fetal gene program [31]. Apart from dephosphorylation by the Ca^2+^- dependent calcineurin phosphatase [24] NFAT is regulated by GSK3β. GSK3β is a cytosolic serine-/threonine kinase which phosphorylates NFAT thereby preventing its nuclear translocation [32]. Here we show for the first time GRK2 dependent phosphorylation of GSK3β. Both, GRK2 overexpression as well as GPCR stimulation using ANG II and PE increased GSK3β phosphorylation. Phosphorylation of GSK3β inhibits its kinase activity and promotes nuclear NFAT activity [32]. Interestingly, GRK2 inhibition via siRNA diminished not only GSK3β-phosphorylation but also reduced nuclear NFAT activity. Inhibition of GSK3β using LiCl resulted in an increase of cell size independent of ANGII treatment or GRK2 inhibition by siRNA. This finding substantiates GSK3β as direct downstream effector of GRK2 such promoting GPCR dependent cardiac hypertrophy. GSK3β upstream regulator Akt was found to be altered in a GRK2 dependent manner. Activity of Akt is regulated by phosphorylation of Ser473 and Thr308 [33]. While phosphorylation of one residue is sufficient for Akt activation, phosphorylation of both residues is required for maximum kinase activity. Stimulation by PE showed increases in Thr308 phosphorylation and increased phosphorylation of Ser473, while ANG II stimulation predominantly regulates phosphorylation status of the Ser473 residue. These differences may be due to higher GRK2 levels following PE stimulation or interactions with other signaling pathways associated with cardiac hypertrophy. However, we have evidence that GRK2 regulates NFAT and induction of cardiac hypertrophy via Akt and GSK3β (Fig 8).

### GRK2-PI3K interaction mediates prohypertrophic effects

The interaction of GRK2 with the Akt activating kinase PI3Kγ, which, if constitutively activated induces hypertrophy, is well described [23]. We therefore hypothesized that the GRK2-PI3Kγ interaction might couple GPCR stimulation to Akt/GSK3β/NFAT via GRK2 induction. In the present study we could demonstrate that GRK2 overexpression not only induces cardiac myocyte hypertrophy but also facilitates GRK2/PI3Ky interaction and promotes nuclear NFAT accumulation. Moreover, pharmaceutical inhibition of PI3K by Wortmannin as well as Akt inhibition by MK220 could abolish all prohypertrophic GRK2 effects and NFAT activation. As PI3Kγ has been shown to be the crucial subunit in mediating cardiac hypertrophy, this indicates the GRK2/PI3Kγ complex as an essential link for GRK2 mediated cardiac hypertrophy (Fig 8).

### GRK2 KO attenuates NFAT activation following transverse aortic constriction

The in vivo relevance of GRK2 dependent NFAT activation could be confirmed by analysis of RCAN expression in GRK2KO mice following TAC operation. The dynamic increase in RCAN expression in WT animals from day 1 to day 4 following TAC correlates well with the time curve of NFAT activation upon TAC reported by Wilkins et al. [24] making it a valuable readout of transcriptional NFAT activity. Importantly, this rise in RCAN expression was almost entirely abrogated in GRK2KO mice, indicating an attenuated NFAT activation in the absence of GRK2.

The importance of GRK2 in cardiomyocyte hypertrophy is supported by a recent study from Kamal et al. [34], which reported to abolish cardiac hypertrophy following TAC by treatment with the Gβγ inhibitor Gallein, known to inhibit GRK2 membrane translocation. Interestingly, both GRK2/PI3Kγ membrane translocation and Akt and GSK3β phosphorylation were decreased in Gallein treated TAC animals hence strongly supporting the results from the present study.

In contrast, the Gβγ inhibitor βARKct, which consists of the GRK2 c-terminal Gβγ binding domain, did not attenuate cardiac hypertrophy following TAC [25, 35]. The observed discrepancies could not only be explained by distinct binding affinities for Gβγ between Gallein and βARKct, but also by potential indirect effects of Gallein treatment on non-cardiomyocytes mediating the antihypertrophic properties. Notably, βARKct contains both the GRK2 binding domains for Gβγ and the majority of the PI3K and Akt binding domains suggesting that specific scaffolding functions of GRK2 could be maintained by this particular GRK2 domain. However, opposing effects regarding the βARKct dependent Akt activation have been described in two studies [36, 37]. In addition, Gαq inhibition was shown to reduce hypertrophic response and PI3Kγ activation upon TAC [16]. Therefore, the interplay of Gαq and Gβγ seems to regulate GRK2 alignment and signaling towards PI3K as downstream effector and disruption of this structural interaction could well explain the antihypertrophic effects of genetic GRK2 ablation.

In conditions like HF or hypertension with chronically increased SNS and RAAS activity and enhanced GRK2 expression [38], GRK2 knockdown can influence GPCR signaling by decreasing its phosphorylation state, which in turn attenuates the subsequent β-arrestin binding. As a consequence, canonical GPCR signaling and GPCR density are maintained.

Of particular interest in this regard are previous reports, which found that PI3K and Akt could be activated in a β-arrestin-dependent manner [39]. β-arrestins serve as multiprotein scaffolds and interact with diverse signaling molecules facilitating non-canonical GPCR signaling. Hence, β-arrestin could be hypothesized to be involved in the GRK2 dependent activation of PI3K/Akt signaling and hypertrophy.

Further investigations will be necessary to clarify whether additional GRK2 binding partners are involved in prohypertrophic GRK2 signaling and to what extend the alignment of GRK2 with other molecules is influencing this.

Furthermore, recent data suggest that GRK2 kinase activity activates nuclear factor 'kappa-light-chain-enhancer' of activated B-cells (NF-κB), thus participating in cardiac hypertrophy [26]. Nevertheless, as GRK2 interacts with a diversity of GPCR and non-GPCR targets, its pro hypertrophic effects are most likely not mediated via a single pathway but rather by modulation of signaling networks resulting in cardiac hypertrophy. Among the pro hypertrophic pathways, GRK2 dependent NFAT activation via PI3Kγ-Akt-GSK3β represents a novel and substantial mechanism.

## Conclusion

In the present study we investigated the role of GRK2 in the promotion of cardiac hypertrophy. Using our established conditional GRK2 mouse line and *in vitro* studies on NRVMs we could demonstrate an essential role of GRK2 in cardiac hypertrophy. In response to GPCR (ANG II and PE) stimulation GRK2 expression rises and initiates the Akt-GSK3β-NFAT prohypertrophic pathway most likely via PI3Kγ-interaction. As cardiac hypertrophy precedes HF, early inhibition of relevant pathways could prevent disease progression. In this regard, GRK2 is a potential novel promising target for treatment of cardiac hypertrophy.

## Supporting information

S1 Fig G-protein coupled receptor kinase 2 knockout (GRK2KO) attenuates cardiac hypertrophy in vivo. Echocardiographic assessment of ventricular remodeling and function. Left ventricular end-diastolic and end-systolic diameter (EDD and ESD), fractional shortening (FS), anterior/posterior wall thickness/diameter (LVAWD and LVPWD) and relative wall diameter before, 3 weeks and 6 weeks after TAC or sham operation in GRK2KO and wild-type (WT) animals. TAC GRK2 KO (red line, n = 18), TAC WT (blue line, n = 13), Sham GRK2KO (black dotted line, n = 6), Sham WT (grey dotted line, n = 8), two-way ANOVA, * P < 0.05 TAC WT vs. TAC GRK2KO.
(TIF)

S2 Fig Induction of fetal gene program under Angiotensin II (ANG II) and Phenylephrine (PE) stimulation is attenuated by G-protein coupled receptor kinase 2 (GRK2) knockdown. Quantitative real-time PCR (RT-PCR) analysis of PE/AngII stimulated neonatal rat ventricular myocytes (NRVMs) with and without siGRK2 treatment. Atrial natriuretic factor (ANF), brain natriuretic peptide (BNP), β myosin heavy chain (βMHC). All values are normalized to calsequestrin (CSQ) mRNA and siRNA scrambled (Scr) treated control group, n = 3-9/group, one-way ANOVA, * P < 0.05, ** P < 0.01, *** P < 0.001.
(TIF)

S1 File Expanded materials and methods section. (DOCX)

S2 File Tabular summary of all data included in the manuscript. (XLSX)
