# Supplementary figures and images for: G protein-coupled receptor kinase 2 promotes cardiac hypertrophy
Source: PLoS One. 2017 Jul 31;12(7):e0182110. doi: 10.1371/journal.pone.0182110 (PMC5536362; doi:10.1371/journal.pone.0182110)

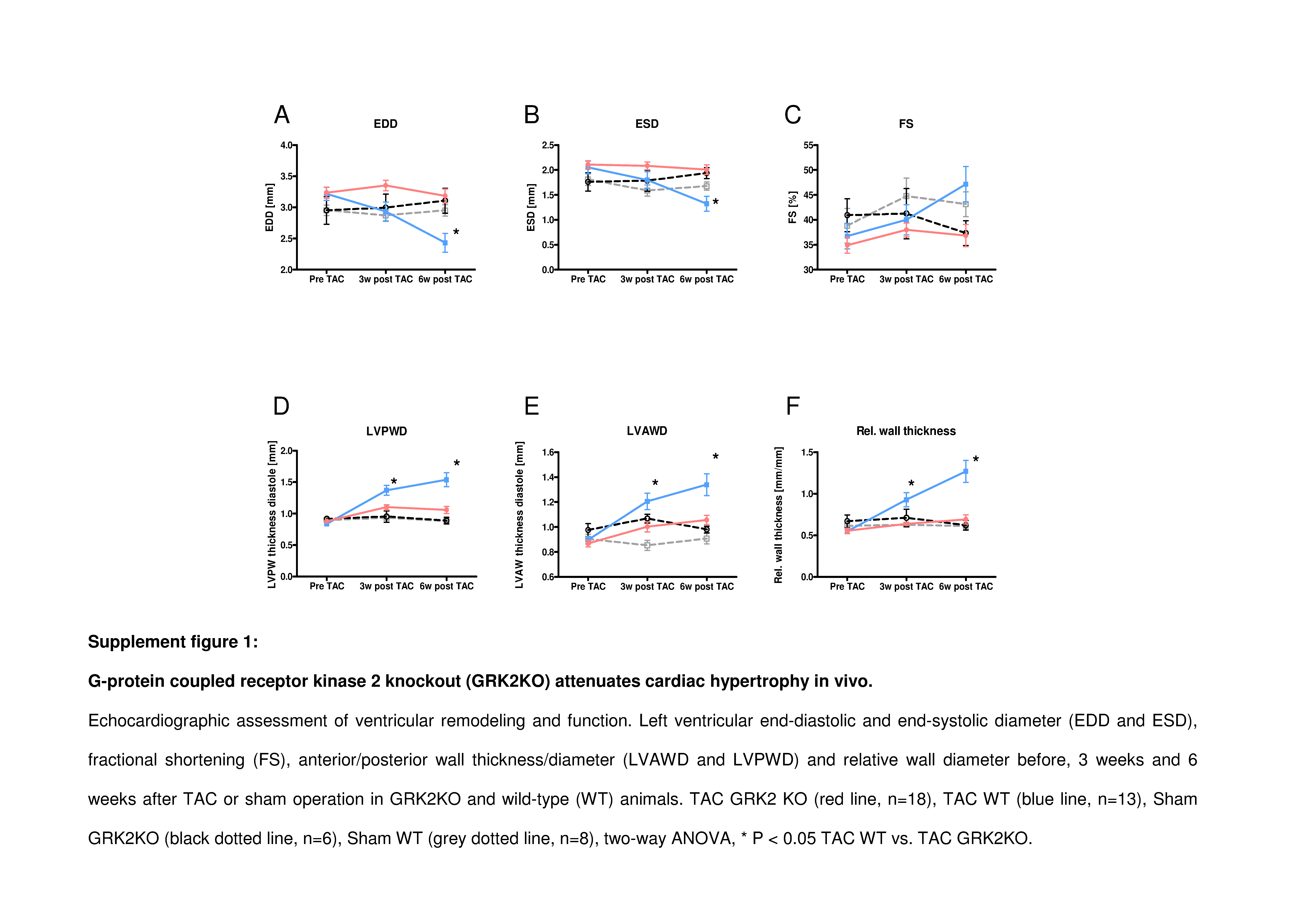

Supplement: S1 Fig — Echocardiographic assessment of ventricular remodeling and function. Left ventricular end-diastolic and end-systolic diameter (EDD and ESD), fractional shortening (FS), anterior/posterior wall thickness/diameter (LVAWD and LVPWD) and relative wall diameter before, 3 weeks and 6 weeks after TAC or sham operation in GRK2KO and wild-type (WT) animals. TAC GRK2 KO (red line, n = 18), TAC WT (blue line, n = 13), Sham GRK2KO (black dotted line, n = 6), Sham WT (grey dotted line, n = 8), two-way ANOVA, * P < 0.05 TAC WT vs. TAC GRK2KO. (TIF) [file pone.0182110.s001.tif]

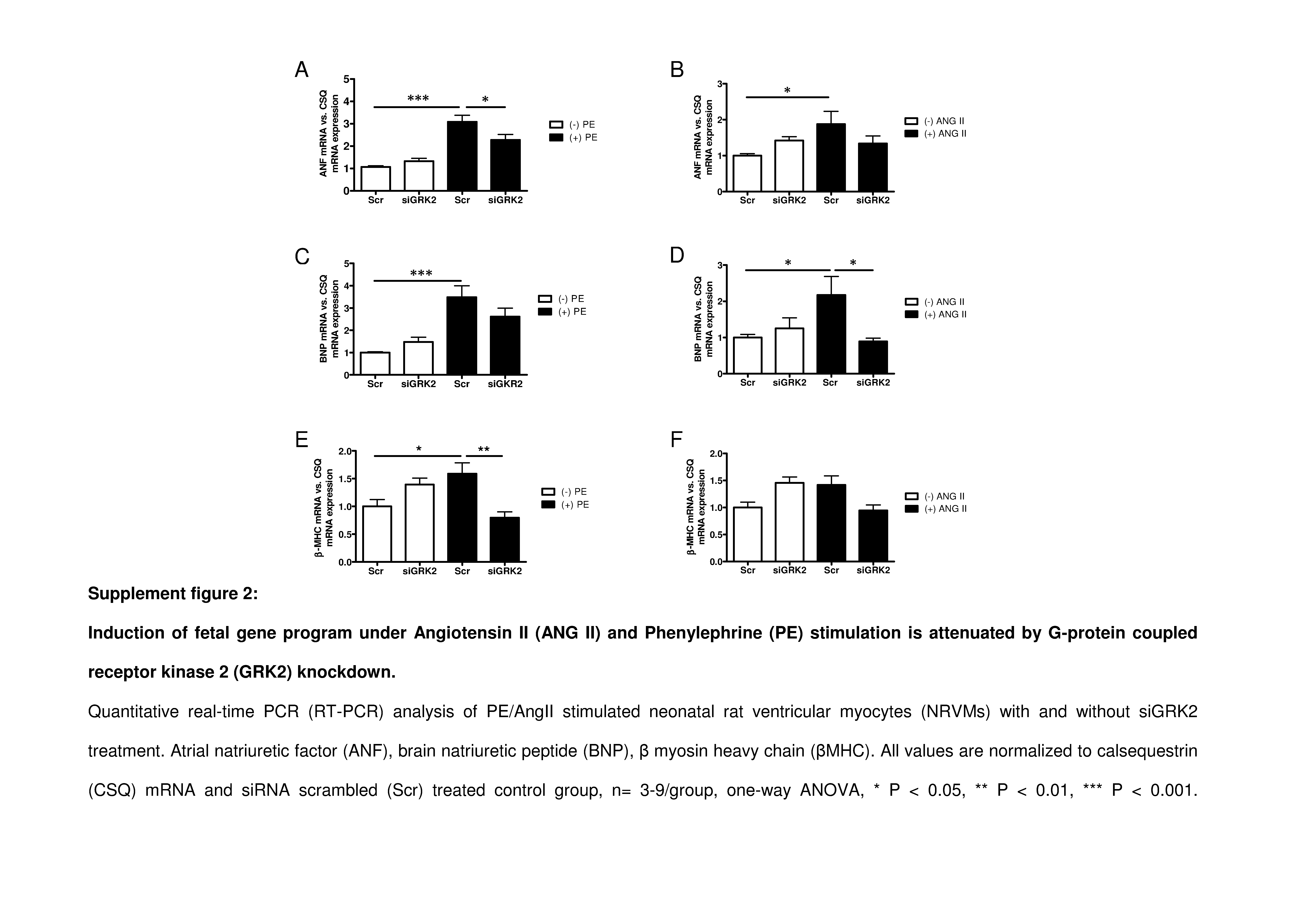

Supplement: S2 Fig — Quantitative real-time PCR (RT-PCR) analysis of PE/AngII stimulated neonatal rat ventricular myocytes (NRVMs) with and without siGRK2 treatment. Atrial natriuretic factor (ANF), brain natriuretic peptide (BNP), β myosin heavy chain (βMHC). All values are normalized to calsequestrin (CSQ) mRNA and siRNA scrambled (Scr) treated control group, n = 3-9/group, one-way ANOVA, * P < 0.05, ** P < 0.01, *** P < 0.001. (TIF) [file pone.0182110.s002.tif]
