# Supplementary material for: G protein-coupled receptor kinase 2 promotes cardiac hypertrophy
Source: PLoS One. 2017 Jul 31;12(7):e0182110. doi: 10.1371/journal.pone.0182110 (PMC5536362; doi:10.1371/journal.pone.0182110)
Supplement: S1 File — (DOCX) [file pone.0182110.s003.docx]

**SUPPLEMENTAL MATERIAL**

**DETAILED MATERIAL AND METHODS**

*Experimental animals*

Conditional mice bearing floxed GRK2 alleles were described previously [1, 2]**.** GRK2KO (alpha myosin heavy chain-Cre-recombinase/GRK2flox/flox, αMHC-Cre/GRK2flox/flox) and wild-type (WT) (GRK2flox/flox) mice were maintained on a C57BL/6 genetic background. All animal procedures and experiments were performed in accordance with the guidelines of the Institutional Animal Care and Use Committee of Thomas Jefferson University. GRK2KO and WT mice were 8-14 weeks of age when entering the study. Unstressed normal mice and mice with TAC were studied. At study end hearts were excised, weighed and snap frozen in liquid nitrogen.

*TAC operation*

TAC operation was performed as described previously [3]. Briefly, mice were anesthetized by inhalation of Isoflurane 5%. Following intubation using a blunt 22-gauge venous catheter mice were connected to a volume-cycled rodent ventilator (MiniVent, Hugo Sachs Electronic, March-Hugstetten, Germany) with a 150 µl tidal volume. Anesthesia was maintained by Isoflurane 1.75%. Via a midline cervical incision trachea, carotid arteries, and aortic arch were exposed. Aortic constriction was induced by tying a 7–0 prolene ligature (Ethicon, Summerville, NJ, USA) against a 27 gauge needle, which was promptly removed to yield a constriction of about 0.4 mm in diameter. Sham-operated animals underwent the same operation except for aortic constriction.

*Echocardiography and Doppler Measurement*

Transthoracic echocardiography was performed as previously described [2]**.** To measure global cardiac function, echocardiography was performed at 8 week old mice before TAC operation, 3 weeks and 6 weeks after TAC by use of a VisualSonics VeVo770 imaging system (VisualSonics Inc., Toronto, Ontario, Canada) with a 12-Mhz scan head in sedated animals (Avertin 250 mg/kg administered intraperitoneally). The internal diameter of the left ventricle was measured in the short-axis view from M-mode recordings in end diastole and end systole. Visual Sonics analysis software was used to calculate ejection fraction and fractional shortening. Doppler measurement of the systolic peak gradient across the TAC site was performed in sedated animals (Avertin 250 mg/kg administered intraperitoneally) 2 days after TAC using the VisualSonics VeVo770 System.

*Isolation of mouse ventricular myocytes*

Adult mouse cardiac myocytes were isolated from sham and TAC operated WT and GRK2KO mice as previously described [2, 4].

*Cell culture*

Primary cultures of neonatal rat ventricular cardiac myocytes (NRVMs) were prepared from 1-2 days old Wistar rats (Charles River, Wilmington, MA, USA). Ventricles were minced and tissue fragments digested by trypsin for 10 minutes. This step was repeated 7 times. After digestion, the cell suspension was centrifuged for 15 minutes at 2000 rpm at 4°C. The supernatant was removed gently and the cell pellet was re-suspended in growing medium, consisting of Dulbecco’s modified Eagle’s medium (DMEM, PAA, Pasching, Austria), supplemented with 10 % fetal bovine serum (FBS, PAA), 1 % Antibiotic P/S (PAA) and 1 % L-Glutamine (Sigma Aldrich, St. Louis, MO, USA). Cells were pre-plated in 6-well plates at 37°C and incubated for 90 min to isolate the fibroblast enriched fraction with myocytes. Remaining non-adherent myocytes were collected and counted manually in a Neubauer chamber. NRVMs were cultured in Medium 199 (Sigma Aldrich, St. Louis, MO, USA) supplemented with 10% (v/v) fetal calf serum (FCS), 100 U/ml penicillin, 100 µg/ml streptomycin, 2 mM L-glutamine, at 37 °C and 5 % CO_2_ humidified atmosphere. After 24 hours FCS was reduced to 0.5% and cultured for 24h before stimulation/transfection.

NRVMs were stimulated either with 10^-7^ M Angiotensin II (ANG II, Merck-Millipore, Darmstadt, Germany) or 10^-4^ M Phenylephrine (PE, Sigma Aldrich, St. Louis, MO, USA) to induce cellular hypertrophy.

*GRK2 knockdown using siRNA*

siRNA molecules were designed and delivered by Ambion (Ambion, Berlin, Germany). Using High Perfect transfection reagents (Qiagen, Venia, Netherlands) siGRK2 (sense *5’ UCA AGU UAC UGG ACA GUG A 3’*, anti-sense *3’ UCA CUG UCC AGU AAC UUG A 5’*) and a non-silencing control (sense *5‘ AGC AUU CAU UCG CGU UGG 3‘*, anti-sense *3‘ CCA ACG CGA AUG AAU GCU 5‘*) were transfected into NVRMs 48h and again 96h after isolation. Transfected cells were recovered for 24h and treated with prohypertrophic stimulants (PE, ANG II) for 24h for RT-PCR analysis or 48h for protein analysis. Knockdown of GRK2 was confirmed by Western blotting.

*Adenoviral GRK2 overexpression*

For these experiments NRCM were isolated as described before and plated into 6 well plates in 10% (v/v) FCS containing medium for 24h then FCS was reduced to 0.5% and cultured for 24h before transduction. Cells were either transduced with an adenovirus harboring harboring LacZ (AdLacZ, MOI 50) or GRK2 (AdGRK2, MOI 50) for 48 hours.

*Kinase inhibition*

For pharmacological inhibition of PI3Kγ, Wortmannin (Sigma Aldrich, St. Louis, MO, USA) was added to the respective well to reach a final concentration of 0.1 µM. For inhibition of Akt, MK-2206 (Santa Cruz, Dallas, TX, USA) was added to the respective well to reach a final concentration of 3 µM.

*Western Blotting*

Western blots were performed as previously described[5]*.* The following antibodies were used for immunoblotting (IB): GRK2 (sc-562, Santa Cruz, Dallas, TX, USA), GSK3β (sc-9166, Santa Cruz), phospho-GSK3β (sc-373800, Santa Cruz), Akt (#9272, Cell Signaling, Danvers, MA, USA), phospho-AKT (Ser473) (#4051, Cell Signaling), phospho-AKT (Thr308) (#2965, Cell Signaling), GAPDH (Merck-Millipore, Billerica, MA, USA). Visualization of Western blot signals was performed with secondary antibodies coupled to Alexa Fluor 680 (Invitrogen, Carlsbad, CA, USA) on an Odyssey infrared imager (LI-COR, Lincoln, NE, USA). Images were processed by Odyssey imaging software. Densitometry scans were carried out in the linear range of detection.

*Immunofluorescence*

The cells were fixed in 3% paraformaldehyde (PFA) and permeabilized using 0.05% Triton-X100 buffer. For actin staining, cells were stained with a monoclonal antibody against α-actinin (1:500, 4°C, Sigma-Aldrich, St. Louis, MO, USA) and mounted in Fluoromount G (Biozol, Eching, Germany). DAPI (4',6-diamidino-2-phenylindol, Invitrogen, Carlsbad, CA, USA) was used for nuclear counterstain. Images were acquired using an Olympus IX81 florescence microscope (Olympus, Hamburg, Germany). Surface area was quantified by capturing the complete boundary using Sigma Scan software (Aspire Software international, Ashborn, Virginia, USA).

*Proximity ligation assay*

The Duolink-PLA assay (Olink, Uppsala, Sweden) was performed according to manufacturer’s protocol with minor modifications regarding antibody concentration. Antibodies for NFATc4 (sc-13036, Santa Cruz, Dallas, TX, USA), NFATc1 (sc-1149, Santa Cruz), GRK2 (sc-18409, Santa Cruz) and PI3Kγ (sc-7177, Santa Cruz) were added at a concentration of 6.7 µg/ml. The fluorescence dots of each ligation, indicating protein-protein interaction, were detected using a Nikon laser scanning confocal microscope (Nikon C2+, Düsseldorf, Germany). Other fluorescence assays were analyzed using an Olympus IX81 florescence microscope (Olympus, Hamburg, Germany). Fluorescence microscope images and intensity signals (green spots) were counted using ImageJ software (National Institute of Health, Bethesda, MD, USA).

*Luciferase assay*

Activity of the nuclear factor of activated T-cells (NFAT) transcription factor was determined by transducing cardiac myocytes with a luciferase reporter linked to a promoter with three NFAT sites (gift from Prof Dr. Johannes Backs, University of Heidelberg, Germany). NRVMs were first transduced with recombinant adenovirus harboring the luciferase reporter (AdLuc, MOI 50) construct for 24 h in 0.5 % serum-reduced culture medium before stimulation with ANG II. Cells were lysed 24 h after stimulation.

For co-transduction experiments NRCM were transduced with AdLuc (MOI 25) and either an AdLacZ (MOI 25) or GRK2 AdGRK2 (MOI 25) for 48 hours. After centrifugation (14000 rpm for 15 min) 200 µl luciferase substrate (E1500, Promega, Madison, WI, USA) were added to the cell lysate and immediately analyzed on a luminometer (Berthold, Bad Wildbad, Germany) according to the manufacturer’s specifications. Luciferase units were normalized to total protein concentration and the respective controls.

*RNA Isolation and quantitative real-time PCR (RT-PCR)*

RNA was extracted using TRIzol reagent (Ambion, Berlin, Germany) from snap frozen tissue (20mg) or cell lysate (1x10^6 cells) according to the manufacturer’s instructions. cDNA was synthesized from 1 µg of total RNA using the iScript cDNA-Synthesis Kit (Bio-Rad Laboratories, Hercules, CA, USA). The quantitative real-time PCR was carried out using iQ-SYBR Green Supermix (Bio-Rad Laboratories) on a MyIQ Single-Color-Real-Time detection system (BioRad Laboratories). Hereby 6.5 µl of diluted cDNA (1/100) were added to a 15µl mixture containing a 1x concentration of iQ SYBR Green Supermix and 100 nM from one of the following primer pairs: ANP forward *5`-TGC CGG TAG AAG ATG AGG TC-3*`, reverse *5`-TGC TTT TCA AGA GGG CAG AT-3`;* BNP forward *5`-CTG AAG GTG CTG TCC CAG AT-3`*, reverse *5`-CCT TGG TCC TTC AAG AGC TG-3`*; βMHC (myosin heavy chain) forward *5`-GCC AAC ACC AAC CTG TCC AAG TTC-3`*, reverse *5`-TGC AAA GGC TCC AGG TCT GAG GGC-3`*; GRK2 forward *5`-CCC TCT CAC CAT CTC TGA GC-3`*, reverse *5`-CGG TTG GGG AAC AAG TAG AA-3`*and calsequestrin (CSQ) forward *5`-TCA AAG ACC CAC CCT ACG TC-3`*, reverse *5`-GGG TCA ATC CAC AAG ATG CT-3`*. RCAN forward *5’-TCC AGC TTG GGC TTG ACT GAG-3’*, reverse *5’-* *ACT GGA AGG TGG TGT CCT TGT C -3’.*

Data were normalized to CSQ as internal standard and the corresponding control group using the *2^–ΔΔCT^* method[6]*.* For each run the melting curve and saturation of amplification cycles were controlled by the use of MyIQ software (version 1.0, BioRad Laboratories).

***Statistics***

All data in the text and figures are presented as mean ± standard error of the mean (SEM). Statistical significance was analyzed using one-way or two-way ANOVA followed by Tukey’s or Bonferroni’s post-hoc test for multiple comparisons if appropriate. Statistical significance between two groups was determined using the two-tailed Student’s t-test or Mann-Whitney-U-test if appropriate. Data analysis was performed using Prism 6 (GraphPad, La Jolla, CA, USA). A P-value *<*0.05 was considered to be statistically significant.

**SUPPLEMENTAL REFERENCES**

1. Matkovich SJ, Diwan A, Klanke JL, Hammer DJ, Marreez Y, Odley AM, et al. Cardiac-specific ablation of G-protein receptor kinase 2 redefines its roles in heart development and beta-adrenergic signaling. Circ Res. 2006;99(9):996-1003. doi: 10.1161/01.RES.0000247932.71270.2c. PubMed PMID: 17008600.

2. Raake PW, Vinge LE, Gao E, Boucher M, Rengo G, Chen X, et al. G protein-coupled receptor kinase 2 ablation in cardiac myocytes before or after myocardial infarction prevents heart failure. Circ Res. 2008;103(4):413-22. doi: 10.1161/CIRCRESAHA.107.168336. PubMed PMID: 18635825; PubMed Central PMCID: PMCPMC2679955.

3. Akhter SA, Luttrell LM, Rockman HA, Iaccarino G, Lefkowitz RJ, Koch WJ. Targeting the receptor-Gq interface to inhibit in vivo pressure overload myocardial hypertrophy. Science. 1998;280(5363):574-7. doi: 10.1126/science.280.5363.574. PubMed PMID: 9554846.

4. Zhou YY, Wang SQ, Zhu WZ, Chruscinski A, Kobilka BK, Ziman B, et al. Culture and adenoviral infection of adult mouse cardiac myocytes: methods for cellular genetic physiology. American journal of physiology Heart and circulatory physiology. 2000;279(1):H429-36. PubMed PMID: 10899083.

5. Premont RT, Macrae AD, Stoffel RH, Chung N, Pitcher JA, Ambrose C, et al. Characterization of the G protein-coupled receptor kinase GRK4. Identification of four splice variants. J Biol Chem. 1996;271(11):6403-10. Epub 1996/03/15. PubMed PMID: 8626439.

6. Livak KJ, Schmittgen TD. Analysis of relative gene expression data using real-time quantitative PCR and the 2(-Delta Delta C(T)) Method. Methods. 2001;25(4):402-8. doi: 10.1006/meth.2001.1262. PubMed PMID: 11846609.
